# Facilitating adaptation to climate change while restoring a montane plant community

**DOI:** 10.1371/journal.pone.0218516

**Published:** 2019-06-20

**Authors:** Christina R. Leopold, Steven C. Hess

**Affiliations:** 1 Hawai‘i Cooperative Studies Unit, University of Hawai‘i at Hilo, Hawai`i National Park, Hawai’i, United States of America; 2 U.S. Geological Survey, Pacific Island Ecosystems Research Center, Kīlauea Field Station, Hawai‘i National Park, Hawai’i, United States of America; Bowling Green State University, UNITED STATES

## Abstract

Montane plant communities throughout the world have responded to changes in temperature regimes by shifting ranges upward in elevation, and made downslope movements to track shifts in climatic water balance. Organisms that cannot disperse or adapt biologically to projected climate scenarios in situ may decrease in distributional range and abundance over time. Restoration strategies will need to incorporate the habitat suitability of future predicted conditions to ensure long-term persistence. We propagated seedlings of three native Hawaiian montane plant species from high- (~2,500 m asl) and low-elevation (~1,900 m asl) sources, planted them in 8 common plots along a 500 m elevation gradient, and monitored microclimate at each plot for 20 weeks. We explored how temperature and precipitation influenced survival and growth differently among high- and low-elevation origin seedlings. Significantly more seedlings of only one species, *Dodonaea viscosa*, from high-elevation origin (75.2%) survived than seedlings from low-elevation origin (58.7%) across the entire elevation gradient. Origin also influenced survival in generalized linear mixed models that controlled for temperature, precipitation, and elevation in *D*. *viscosa* and *Chenopodium oahuense*. Survival increased with elevation and soil moisture for *Sophora chrysophylla*, while it decreased for the other two species. Responses to microclimate varied between the three montane plant species; there were no common patterns of growth or survival. Although limited in temporal scope, our experiment represents one of the few attempts to examine local adaptation to prospective climate scenarios and addresses challenges to restoration efforts within species’ current ranges.

## Introduction

There is a wealth of evidence to support climate-induced shifts in plant species distributions [1–3]. Montane plant communities in widely separated intact natural environments of the world have responded to changes in precipitation and temperature regimes by shifting ranges upward in elevation, moving upslope over 20 m per decade in some instances [2]. Reduced evapotranspiration rates in cooler climate zones at higher elevation may compensate for less precipitation and more extreme temperatures within species’ former ranges. Plants with short generation times and faster population turnover, such as grasses, may be able to quickly disperse upward [2]; however, longer-lived plants that mature more slowly may suffer ‘migration lag’ [4] and consequently fail to keep pace with the rising elevation of climate zones [5]. Alternatively, changes to precipitation patterns in some cases have resulted in downslope movements both within and beyond a species’ range [6, 7]. Crimmins et al. [7] documented downslope movement across species’ ranges, supporting the idea that entire populations are shifting in response to changes in water availability caused by climate change, as opposed to responding to temperature. Another cause of downslope movement may be novel inter-species competition caused by competitive release [8, 3]. HilleRisLambers et al. [9] found both; seedlings were limited by competition at lower range limits, while climate effects were observed at upper range limits for adults and saplings. In either scenario montane populations are predicted to become more fragmented and isolated, increasing risk of local extinctions caused by stochastic events [10, 8].

Organisms that cannot disperse or adapt biologically in situ to rapid environmental changes may decrease in distributional range and abundance. Fragmented forest habitats may have little gene flow due to limited seed dispersal, further reducing species’ ability to adapt naturally. One potential management approach is to transplant conspecifics from low-elevation locations to higher-elevation zones. Translocating species to higher-elevation zones may encourage evolutionary change by moving climate compatible variants to more appropriate zones faster than they can disperse naturally [11, 12]. Alternatively, moving conspecifics downslope may enhance survival, as plants may track climatic water balance from moister air at warmer temperatures. Correctly predicting suitable climatic conditions for plant populations is critical for maximizing restoration and revegetation efforts.

Provenance studies are relatively common in silviculture, and many reciprocal planting studies have investigated how seed origin influences growth and vigor across a species’ range [13]. Local provenancing presumes local plant genotypes to be better adapted to an environment than genotypes from other areas, while admixture provenancing mixes seeds from multiple origins to avoid inbreeding depression. Bucharova et al. [11] introduce regional admixture provenancing, yet another method for sourcing seeds. Aside from model species, provenance trials are not typically conducted within the context of restoration [14], and it is generally accepted that seeds for revegetation projects should be sourced as locally as possible. However, biotic conditions are predicted to change across much of the world, particularly across elevation gradients, although shifts will not occur linearly [10]. Thus, local seed-sourcing, particularly for species with long-generation times, may become an out-dated and potentially ill-suited approach [15]. Seed transfer zones, or geographic areas considered safe to move seeds within and not risk maladaptation [16], have been generally described in the literature for continental species [17]. However, such maps are not transferable in mountainous tropical regions where habitat heterogeneity exists over short geographic distances due to dramatic elevation, precipitation, and edaphic gradients.

Mountain parklands are among the most degraded ecosystems in Hawai‘i [18]. Centuries of adverse land use practices and the proliferation of introduced invasive species have caused deforestation, fragmentation, and isolation in native montane plants, disrupting biological connectivity between high-elevation subalpine woodlands and lower-elevation montane wet and mesic forests. Restoration efforts have largely included sourcing seeds from nearby areas when possible, although provenance is not typically tracked with restoration success metrics. Several native plant species in mountain parkland ecosystems are found naturally over a broad range of elevation, but may become range-restricted if environmental conditions shift rapidly as a consequence of climate change [19]. Restoration efforts that include experimentation with multiple provenances may benefit these degraded systems, and link seed origin to outcome.

Our objective was to determine if important native Hawaiian montane plant species may benefit from enrichment with seeds from climatically appropriate sources to enhance survival, growth, and adaption to changing precipitation patterns by relocating conspecifics to more favorable climate regimes. Native montane species were largely absent from this mountain parkland system in need of restoration; we sought to identify local sources of each species from appropriate climate zones to increase tolerance to contemporary and future climate conditions, and connectivity between existing high-elevation subalpine woodlands and lower-elevation montane wet and mesic forests. Given the pattern of findings that plant species are moving upslope with warming temperatures, we hypothesized that plants from a lower-elevation forest ecotype would have higher rates of survival and growth compared to high-elevation forest conspecifics when grown in common plots along an elevation gradient. Alternatively, we sought to determine if seeds from a high-elevation forest ecotype would outperform those from a low-elevation origin due to more favorable precipitation conditions when planted along the same elevation gradient. In addition to climate, we also considered if distance from seed origin was related to plant survival and growth.

## Methods

### Study site

**Fig 1:**
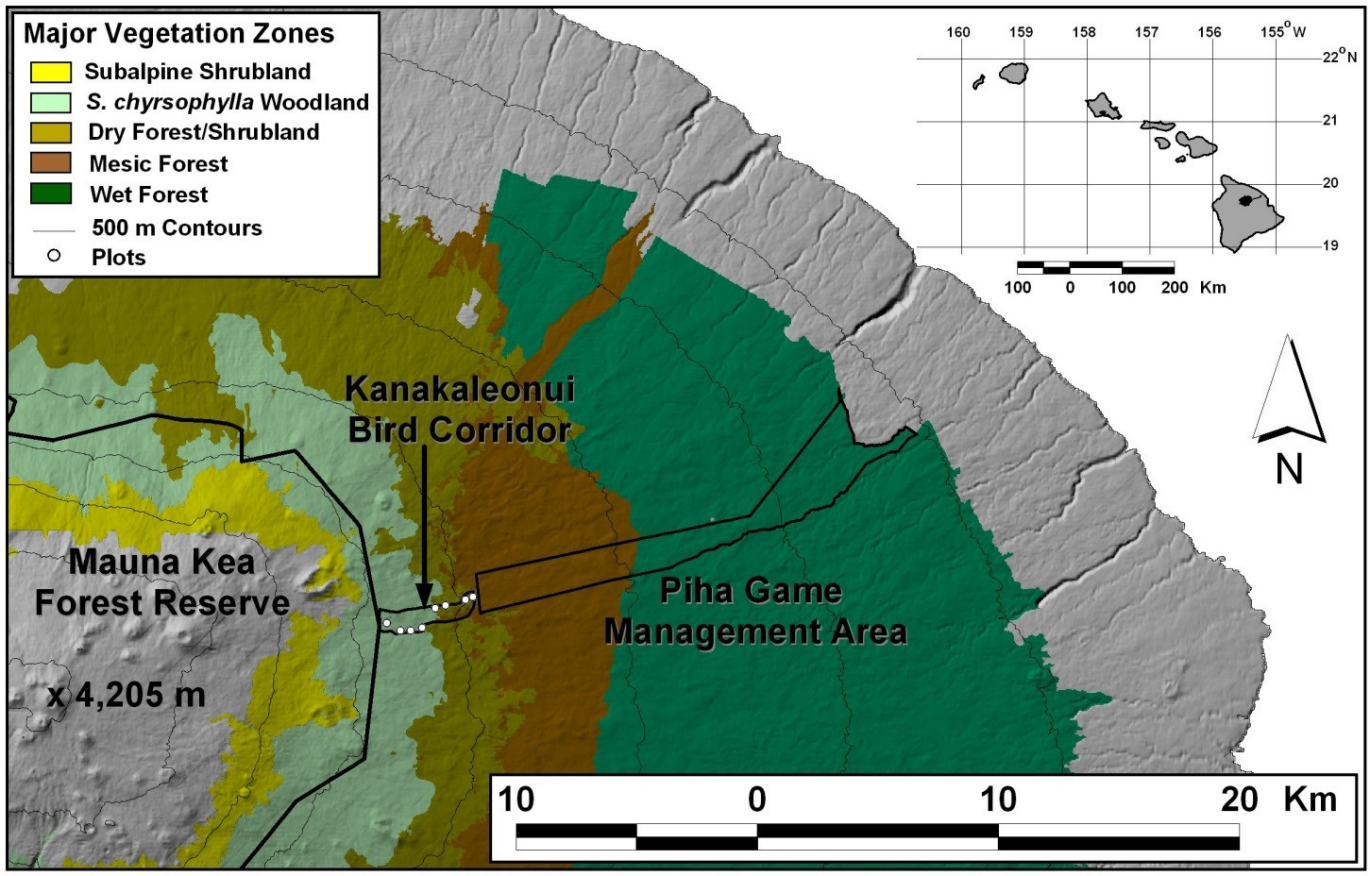
Kanakaleonui Bird Corridor (KBC) on Hawai‘i Island. Seedlings of three native montane species from high- and low-elevation origin were planted in eight plots and microclimate was monitored along a 500 m elevation gradient at approximately 60-m intervals in 2016.

Kanakaleonui Bird Corridor (KBC), which lies between 2,000 and 2,500 m asl on the east slope of Mauna Kea, Hawai‘i Island (19° 15’ 58” N, 155° 21’ 2” W), is a culturally important area in need of ecological restoration (Fig 1). KBC experienced over 100 years of cattle grazing, feral pig activity and logging prior to fencing in 2007, and is now considered severely degraded. Although introduced herbivorous mammals have been removed, invasive pasture grasses such as *Cenchrus clandestinus* and *Holcus lanatus* continue to dominate the mountain parkland ecosystem. Appropriate high- and low-elevation sources for seeds of important native plant species were available from the adjacent Mauna Kea Forest Reserve (>2,500 m asl) and Piha section of Hilo Forest Reserve (<2,000 m asl). Microclimate monitoring and outplanting plots were selected along a 500 m elevation gradient each approximately 60 m apart in elevation within KBC. Plot locations were identified using a GIS and assessed to ensure they consisted of substrate similar to other plots, and relocated as necessary so that microclimate variables constituted the primary difference between sites. All plots lacked canopy cover. Rainfall ranged from 2,085 mm annually at the low-elevation boundary to 985 mm annually at the high-elevation boundary [20].

### Microclimate monitoring

Eight HOBO (Onset Computer Corporation, Bourne, Massachusetts) micro station data loggers (H21-002) were installed at elevation intervals of approximately 60 m within KBC on 16 and 18 December 2014. Each data logger was equipped with two sensors to monitor: 1) temperature and relative humidity (S-TMB-M002), and 2) soil moisture (S-SMC-M005). Temperature and humidity sensors were installed at a height of 1 m above ground at each station, and soil moisture probes at a depth of approximately 10 cm. Data loggers were programmed using HOBOware supporting computer software to store average values at five-minute intervals to allow sufficient battery life and memory storage. Stations were inspected monthly and temperature, relative humidity, and soil moisture data were downloaded. Two stations damaged by feral pigs in January 2015 were repaired and all stations were fenced to exclude large animals in March‒April 2015. Two soil moisture sensors malfunctioned during 1-month periods and were replaced in April and August 2016. We summarized microclimate data by monthly minimum, maximum, and average values for analyses.

### Seed collection and plant propagation

We collected seeds from approximately 10 plants of eight native tree and plant species in the Mauna Kea Forest Reserve (high elevation), and the Piha and Laupahoehoe Sections (low elevation) of the Hilo Forest Reserve (Fig 1). Approximately 8,000 seeds were collected and sent to Maui Native Nursery, LLC for propagation in June 2015. However, only three species were successfully propagated: *Chenopodium*. *oahuense*, *Dodonaea*. *viscosa*, and *Sophora*. *chrysophylla*. Seeds from low-elevation mesic/wet forest were not available for *D*. *viscosa*, and instead were collected from a similar elevation (1900 m asl), but from an area with climate conditions similar to the high-elevation seed source. No aspect of this study involved protected or endangered species.

### Site preparation and outplanting

Seedlings were planted at plots near the eight microclimate monitoring stations. Plots approximately 20 x 20 m in size were sprayed with imazapyr herbicide to kill invasive grasses in July‒August 2015, and again with glyphosate herbicide in January 2016. A total of 322 liters of herbicide mixture was used for the first round of grass control, and a follow-up effort used another 116 liters. Seedlings of three native plant species were hardened in a greenhouse at KBC beginning October 12, 2015: 480 *C*. *oahuense*, 350 *D*. *viscosa*, and 115 *S*. *chrysophylla*. Nineteen *C*. *oahuense*, 22 *D*. *viscosa*, and two *S*. *chrysophylla* seedlings died before being outplanted. Seedlings of each species were equally distributed for outplanting among all eight plots, and planting order within plots was randomized. Number of seedlings planted, by species and plot, is available in S1 Table. All seedlings were outplanted among the eight study sites February 29–March 2, 2016. Seedlings were planted approximately 1 m apart, thoroughly watered, coconut core substrate added when necessary, and amended with Nutricote 13-11-11 fertilizer. Seedling height was measured to the nearest mm in the greenhouse prior to out-planting, and three times after out-planting: during March three weeks after outplanting, June and July 2016. March measurements were used as the initial height value in subsequent analyses. While not initially part of the experiment design, invasive grasses and weeds were manually removed from plots throughout the 2016 growing period to minimize seedling mortality from competition. Heavy herbivory and substantial seedling mortality was caused by non-native game birds at Plot 2, and data from that plot were eliminated from statistical analyses.

### Analyses

We compared overall seedling survival across and within each species, by origin (high or low-elevation) using chi-square tests. We used linear mixed models to analyze factors associated with plant growth, and generalized linear mixed models to analyze the effect of factors associated with survival. We created separate growth and survival models for each species to maintain comparability across model types, and because multi-species models did not converge. Separate models for each species also allowed model effects such as interactions to be more easily interpreted. We included interactions of origin with climate variables which would indicate that the origin of seed is an important consideration for restoration. An origin by elevation interaction effect, i.e., low origin seedlings do well in low but not high elevations and vice versa, may indicate origin matching elevation, as opposed to greater survival and growth in only low-elevation origin plants.

Predictors included in mixed model analyses for both the growth and survival models were the same: plot as a random effect, seed origin (high or low elevation), plot elevation, temperature range (maximum–minimum values), and mean monthly soil moisture. Monthly minimum, maximum, and mean temperatures, mean monthly relative humidity, and seedling distance from origin as well as several interactions were considered, but not included as model parameters after correlation tests. Mean temperature and relative humidity lacked variation between plots and were eliminated as model predictors. We used Pearson’s correlation statistic to assess relationships between continuous predictors, and removed several to reduce collinearity. Elevation by origin and distance from origin were strongly correlated (r = 0.77–0.83 for all categories). We eliminated distance from origin from further model efforts in order to more explicitly assess origin along the elevation gradient. Minimum temperature was eliminated from consideration due to its strong negative correlations to elevation (r = -0.77) and temperature range (r = -0.68). Maximum temperature was eliminated as a factor due to a high correlation with temperature range (r = 0.91). We retained temperature range to serve as a proxy for exposure. There was a moderate negative correlation between elevation and soil moisture (r = -0.62). Values for microclimate variables were assigned based on the time period an individual seedling was in the ground, i.e., plants that died between the June and July measurements were assigned an average of the variable using March‒June data, while those surviving the duration of the experiment were assigned data from March‒July.

Data for growth included an individual slope of growth per day (growth rate index) for each seedling: growth (mm) ~ days since initial height measurement. Data were weighted based on the number of days between measurements. Seedlings with measurements for March–July received a weight of 2.0, those with March–June 1.75, and those with June–July a weight of 1.0.

Mixed effect models were used to account for variation due to microclimatic variables not captured by fixed effects. Plot was treated as a random effect and elevation was included as a fixed effect despite their correlation to explain additional variation in models. All data analyses were conducted in R 3.3.1 [21]. We analyzed seedling survival using logistic regression models (generalized linear mixed models with logit link function) due to the binary nature of the data. Growth models were fitted using linear mixed effect models [22] and restricted maximum likelihood (REML) due to unbalanced samples. We used AICc reverse stepwise model selection for both growth and survival models [23]. Fixed and random effects were evaluated using packages ‘sjstats’ and ‘sjPlot’ [24, 25].

## Results

### Survival

**Fig 2:**
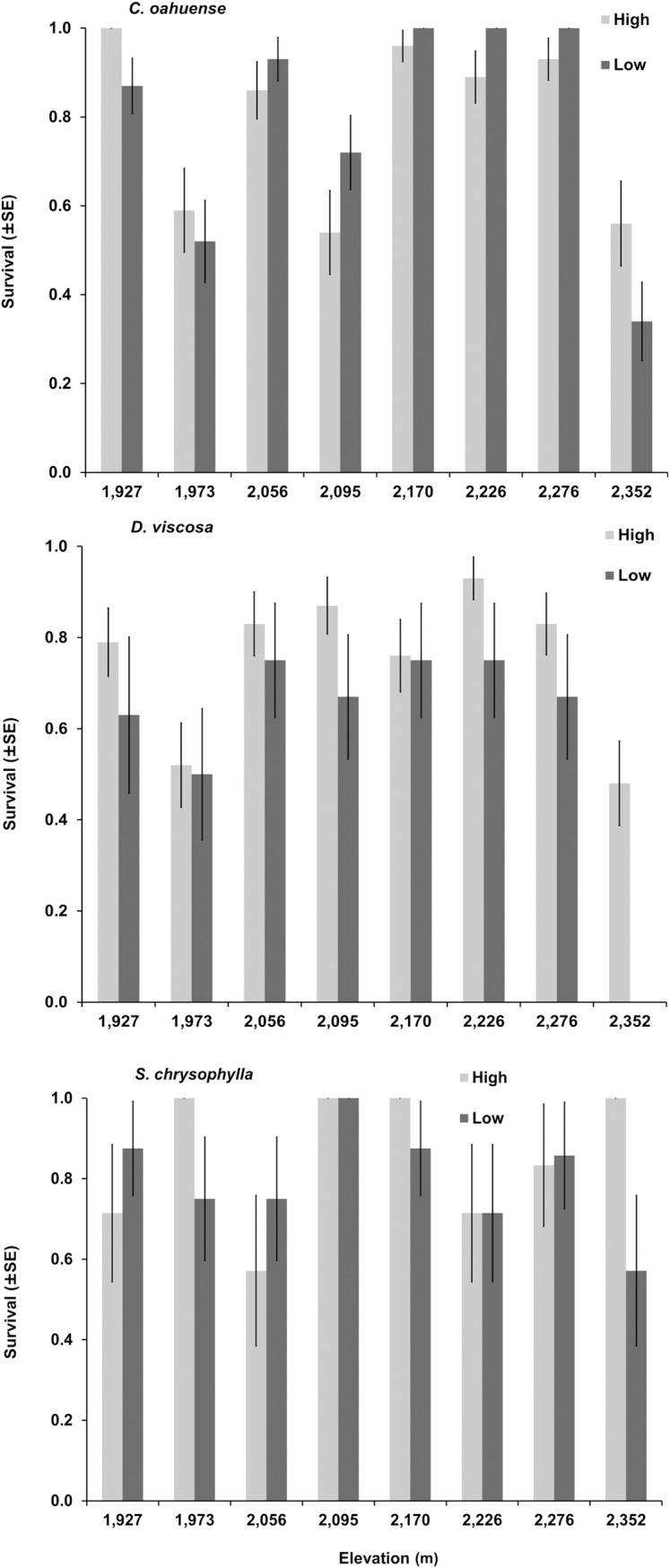
Survival (±SE) of 896 seedlings over a 20 week period. Seedlings from high- and low-elevation origin were planted along an elevation gradient in eight plots at Kanakaleonui Bird Corridor, Hawai‘i Island.

Plant survival was variable between plots (Fig 2; Table in S1 Table), generally high among all three plant species, and overall survival of species did not differ between high- and low-elevation origins (Table 1). Survival of high-elevation origin *Dodonea viscosa* (75.2%) was significantly greater than that of low-elevation origin (58.7%).

**Table 1:** Chi-square tests of difference in survival between three species of seedlings grown from high and low-elevation sources at Kanakaleonui Bird Corridor, Hawai‘i Island.

Species	*χ*^*2*^	*P*
*Chenopodium oahuense*	0.009	0.924
*Dodonaea viscosa*	8.672	0.003
*Sophora chrysophylla*	1.900	0.168
All	0.963	0.326

In logistic regression mixed effect models, plant survival models varied across species (Table 2). Although survival of all three species was predicted by elevation and soil moisture, the relationship was variable for all species (Table 3). The random effect of plot did not explain variation in highest-ranked models (ICC < 0.001) for *D*. *viscosa* and *Sophora chrysophylla* (Table 4). However, plot was retained to account for micro-variation between plots in models, and to maintain comparability across species.

**Table 2:** Summary of effects in highest-ranked models of montane plant species, *Chenopodium oahuense*, *Dodonaea viscosa*, and *Sophora chrysophylla*.

Model	Predictors
Survival	
*C*. *oahuense*	Elev + Temp + Water + Origin + Temp*Origin + Plot
*D*. *viscosa*	Initial height + Elev + Temp + Water + Origin + Elev*Origin + Plot
*S*. *chrysophylla*	Initial height + Elev + Water + Plot
Growth	
*C*. *oahuense*	Initial height + Dist + Elev+ Origin + Elev*Origin + Plot
*D*. *viscosa*	Plot
*S*. *chrysophylla*	Plot

Plot was specified as a random effect in all models. Model predictors included initial seedling height (Initial height), seedling origin (Origin), plot elevation (Elev), temperature range (Temp), average soil moisture (Water), and the interactions of Origin with Elev, Temp, and Water, respectively.

**Table 3:** Direction of effects on plant survival by species and origin of seed.

	Plant Species					
	*C*. *oahuense*		*D*. *viscosa*		*S*. *chrysophylla*	
Fixed effect	High	Low	High	Low	High	Low
Initial height	–	–	↑	↑	↑	↑
Seed Origin	–	↑	–	↓	–	–
Elevation	↓	↓	↓	↓↓	↑	↑
Temperature	↑	↑↑	↑	↑	–	–
Water	↓	↓	↓	↓	↑	↑

Reference category was high elevation origin. Initial seedling height, origin of seed (high or low elevation), plot elevation, temperature range, and average soil moisture, as well as the interaction of origin with elevation, temperature, and origin, respectively, were fixed effect terms included in modeling.

An ↑ indicates a positive influence on plant growth while an ↓ indicates a negative influence on plant growth. Dash indicates that the term was not a factor in the highest-ranked linear mixed model. A double arrow is displayed when the magnitude of the effect varied with seed origin.

**Table 4:** Model output values, by species, for each best model predicting seedling survival (Tables in S2–S4 Tables).

	Plant Species								
	*C*. *oahuense*			*D*. *viscosa*			*S*. *chrysophylla*		
	*OR*	*CI*	*p*	*OR*	*CI*	*p*	*OR*	*CI*	*p*
*Fixed Effect*									
(Intercept)	8.75	4.16 – 18.40	<0.001^*a*^	5.05	3.39 – 7.50	<0.001^*a*^	35.67	5.94 – 214.33	<0.001^*a*^
Initial height				1.95	1.27 – 3.01	0.002	3.92	1.27 – 12.11	0.018^*a*^
Seed origin (Low)	1.70	0.86 – 3.37	0.125	0.52	0.27 – 1.01	0.055			
Elevation	0.25	0.12 – 0.53	<0.001^*a*^	0.61	0.41 – 0.91	0.015^*a*^	2.74	1.10 – 6.80	0.030^*a*^
Temperature	1.05	0.49 – 2.24	0.898	1.66	1.21 – 2.27	0.002^*a*^			
Water	0.23	0.11 – 0.50	<0.001^*a*^	0.66	0.46 – 0.95	0.025^*a*^	2.69	1.03 – 7.07	0.044^*a*^
Elevation*Origin				0.53	0.26 – 1.09	0.085			
Temperature*Origin	2.63	1.12 – 6.17	.027^*a*^						
Water*Origin									
*Random Effect*									
τ_00, Plot_	0.459			0.000			0.000		
N_Plot_	7			7			7		
ICC_Plot_	0.122			0.000			0.000		
Observations	393			280			96		
Deviance	241.739			272.540			77.786		

Initial seedling height, origin of seed (high or low elevation), plot elevation, temperature range, and average soil moisture, as well as the interaction of origin with elevation, temperature, and soil moisture, respectively, were fixed effect terms included in modeling. Odds ratio values (*OR*) indicate an increased probability of survival when values >1. A confidence interval (*CI*) and p-value (*p*) are presented for each fixed effect. Random effect interpretation includes: tau.00, Kendall’s tau between-group variance of the random slope; and the intra-class correlation coefficient (ICC), the amount of overall variation that can be explained by the grouping of Plot [24].

^*a*^ indicates statistical significance at the 0.05 level.

*Chenopodium oahuense* survival probability was inversely related to soil moisture and elevation, with approximately four times greater probability of survival in locations with low soil moisture and elevation respectively (Table 4; Table in S2 Table). Survival increased slightly with temperature range for all seedlings, and 163% for seedlings of low elevation origin (w_*i*_ = 0.31). The random effect of plot explained a large proportion of model variation (ICC = 0.459).

Survival of *D*. *viscosa* was influenced by all microclimate variables and initial seedling height (Table 4; Table in S3 Table). Seedlings from high-elevation origin had a 92% greater probability of survival overall. Soil moisture and elevation were inversely related to seedling survival with seedlings at lower elevation having a 64% greater probability of survival, with an even lower survival probability for seedlings from low-elevation origin planted at high elevations. Initial height increased seedling survivorship probabilities by 95%, and increasing range of temperature by 66% (w_*i*_ = 0.37).

Initial seedling height, elevation, and soil moisture all positively influenced *S*. *chrysophylla* survival. The effect of origin was not informative in the highest-ranked model alone or as an interaction term (w_*i*_ = 0.32; Table in S4 Table). Taller initial seedling heights increased survival probability by 292%, while seedlings at higher elevations and those with high soil moisture increased survival probability by 174% and 169%, respectively.

### Growth

**Fig 3:**
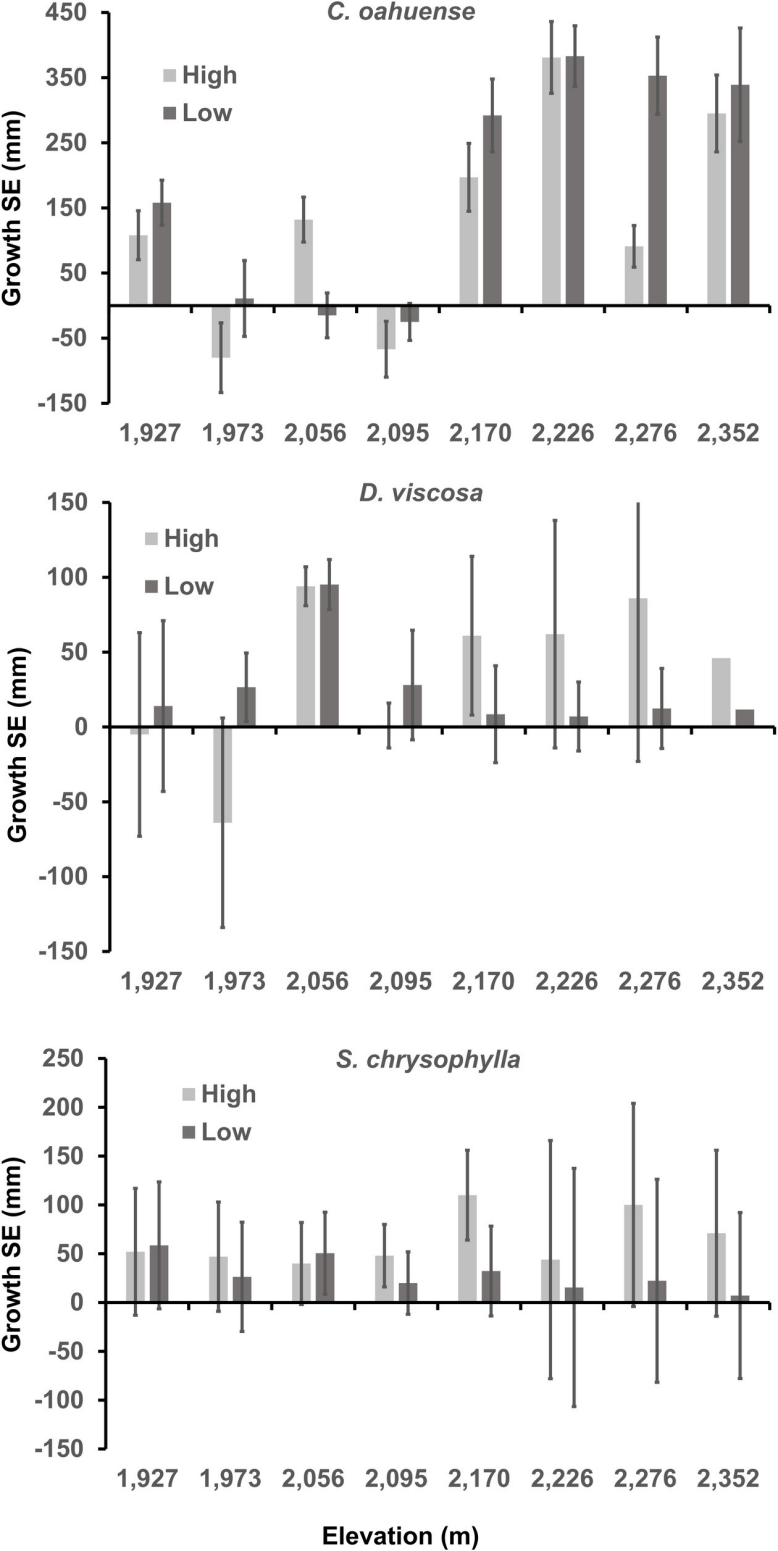
Average growth (±SE) of 744 seedlings over a 20 week period. Seedlings from high- and low-elevation origin were planted along an elevation gradient in eight plots at Kanakaleonui Bird Corridor, Hawai‘i Island.

Plant growth within each species varied dramatically by plot (Fig 3). Predictors of plant growth in linear mixed models varied between species (Table 5), and for two of the three species, the base model held the most weight (Tables in S5–S7 Tables). Elevation and soil moisture were not predictive of plant growth in any top models (Table 6). The model predicting growth per day for *C*. *oahuense* indicated that initial seedling height negatively influenced growth over time, and that as temperature range increased, growth rate increased for seedlings from low elevation origin. The random effect of plot explained approximately 18% of the model variance (ICC = 0.184).

**Table 5:** Direction of fixed effects on plant growth over time, by species and origin of seed.

	Plant Species					
	*C*. *oahuense*		*D*. *viscosa*		*S*. *chrysophylla*	
Fixed Effect	High	Low	High	Low	High	Low
Initial height	↓	↓	–	–	–	–
Seed Origin	–	↑	–	–	–	–
Elevation	–	–	–	–	–	–
Temperature	↓	↑	–	–	–	–
Water	–	–	–	–	–	–

Initial seedling height, origin of seed (high or low elevation), plot elevation, average temperature, and average soil moisture, as well as the interaction of origin with elevation, temperature, and origin, respectively, were fixed effect terms included in modeling.

An ↑ indicates a positive influence on plant growth while an ↓ indicates a negative influence on plant growth. Dash indicates that the term was not a factor in the highest-ranked linear mixed model.

**Table 6:** Model output table, by species, for each best model predicting seedling growth over time (Tables in S5–S7 Tables).

	Plant Species								
	*C*. *oahuense*			*D*. *viscosa*^*a*^			*S*. *chrysophylla*^*a*^		
	*B*	*CI*	*p*	*B*	*CI*	*p*	*B*	*CI*	*p*
*Fixed Effect*									
(Intercept)	0.29	-0.24 – 0.83	0.303						
Initial height	-0.41	-0.53 – -0.28	<0.001^*b*^						
Seed origin (Low)	0.37	0.14 – 0.59	0.007^*b*^						
Elevation									
Temperature	-0.06	-0.59 – 0.47	0.826						
Water									
Elevation*Origin									
Temperature*Origin	0.31	0.09 – 0.52	0.016^*b*^						
Water*Origin									
*Random Effect*									
σ^2^	2.125								
τ_00, Plot_	0.480								
N_Plot_	7			7			7		
ICC_Plot_	0.184			0.083			0.024		
Observations	345			234			82		
R^2^	0.332								

Initial seedling height, origin of seed (high or low elevation), plot elevation, temperature range, and average soil moisture, as well as the interaction of origin with elevation, temperature, and soil moisture, respectively, were fixed effect terms included in modeling. Slope (*B*) with a confidence interval (*CI*) and p-value (*p*) are presented for each fixed effect. Random effect interpretation includes: sigma-squared, the within-group variance; tau.00, Kendall’s tau between-group variance of the random slope; and the intra-class correlation coefficient (ICC) is the amount of overall variation that can be explained by the grouping of Plot [24].

^*a*^ Base model was best for predicting seedling growth.

^*b*^ Indicates statistical significance at the 0.05 level.

The highest-ranked model for *D*. *viscosa* was the base model, with a moderately strong model weight (w_*i*_ = 0.52; Table in S6 Table). The second-ranking model contributed substantially to account for most of the remaining weight among those in the model suite (w_*i*_ = 0.42), which included the negative influence of soil moisture on seedling growth. The random effect of plot explained 8.3% of model variation (ICC = 0.083) in the base model.

The base model, and one including initial seedling height, were tied for highest-ranked models predicting growth of *S*. *chrysophylla* seedlings, each holding nearly half of the model weight (Base w_*i*_ = 0.45, Height w_*i*_ = 0.45; S7 Table). The random effect of plot explained 1% of the variation in plant growth (ICC = 0.010), indicating that a mixed model may not have been more informative than a traditional linear model for predicting plant growth in this case. However, the random effect of plot was retained to account for micro-variation between plots in the model, and to maintain model comparability across species.

## Discussion

The scale of our experiment did not capture population-level differences that would be useful to exploit for restoration strategies for two of the three species included. Overall, seedlings from mesic-wet forest and subalpine shrubland environments did not demonstrate short-term differences in growth and survival. Although seed sources for *C*. *oahuense* and *S*. *chrysophylla* differed by 500 m in elevation and varied > 1,000 mm of annual rainfall, they were ≤4 km apart in distance. Perhaps the scale at which seeds were sourced encompassed a ‘local’ provenance. While plots spaced ~60 m apart in altitude was a small interval, there is evidence for climatic conditions affecting montane plant vigor across similarly-spaced plots. Inouye [26] documented flower bud death caused by frost differed >20% between neighboring plots 12 m apart in elevation, and more dramatic differences across 90 m elevation. Although not explicitly tested, sufficient gene flow may be retained in the common species of *C*. *oahuense* and *S*. *chrysophylla* given the relatively small distance separating populations, and further studies examining the genotypic variation within seed sources would yield complementary insights. In contrast, *D*. *viscosa* had significantly greater survival in seedlings from high-elevation origin than those from low-elevation origin, and the source of these low-elevation seedlings was approximately 30 km from KBC on the southwestern slope of Mauna Kea. Furthermore, furthest-sourced *D*. *viscosa* seedlings had the lowest survival in our experiment. It is possible that population-level variation exists across this broader 30 km distance, an order of magnitude greater than that of seeds sourced for *C*. *oahuense* and *S*. *chrysophylla*.

Most of the variation between sites may have been captured by the measurements of temperature range and moisture, both of which influenced seedling survival. Survival models for *C*. *oahuense* and *D*. *viscosa* indicated that microclimate conditions at highest elevation locations were unfavorable generally, despite origin. Survival was similar across plots for *S*. *chrysophylla*, the dominant subalpine tree species, for plants of both origins. In a meta-analysis by Leimu and Fischer [13], distance and habitat variability were not demonstrably influential in reciprocal planting experiments; population size of origin plants was the strongest predictor of provenance-trial success. We documented results along a similar vein: despite dramatic environmental differences in seed origin, factors influencing seedling survival acted in the same direction for each species, albeit with varying magnitudes of effect in some instances.

Larger-scale climate changes such as the trade wind inversion that limits upward movement of prevailing moisture-laden trade winds is an especially important influence of temperature and rainfall in montane environments of Hawai‘i [27]. Weather variability, warming, and rainfall patterns are affected within the inversion layer [27]. Crausbay and Hotchkiss [28] found that the mean trade wind inversion strongly influenced species assemblages on Maui. The inversion layer on Hawai‘i Island has a diurnal base height range of 2,201–2,255 m, which included three of our study plots in the upper portion of KBC. Documented effects of increased number of trade wind inversion days, and their associated drier and warmer conditions have increased mortality and decreased recruitment of Haleakalā silverswords (*Argyroxiphium sandwicense* subsp. macrocephalum), particularly near range limits, over the last decade [29]. There is evidence that the inversion layer has been decreasing in elevation, and the lifting condensation level is increasing, which may result in reduced cloud thickness, rainfall, and overall size of the rain-belt on mountain slopes [30]. This shift could reduce the extent of wet and mesic forests at their upper-elevation limits, and favor drier mountain parkland communities. Restoration strategies may benefit from incorporating these future scenarios. Provenance trials within restoration plantings would be relatively simple to conduct and could determine if more drought- and exposure-tolerant variants are appropriate for these transition zones.

The short-term nature of our experiment precluded meaningful findings about enhancing genetic enrichment to build resiliency in restoration strategies; however, limited as is was, it represents one of the few attempts to examine the effects of local climate adaptation within species’ current ranges while conducting restoration experiments in the presence of competition and disturbance from invasive species. Other studies have examined upper-elevation extension of species ranges by observation [31–33] or manipulation [34–36] within native systems, but have not addressed continued persistence within existing species’ ranges, which will be essential for understanding responses of broadly distributed species (but, see review by Godefroid et al. [37]). Reciprocal and multi-site provenance trials are also often conducted within relatively intact systems [13] in contrast to the degraded mountain parkland in our study [18, 38]. Further studies to robustly test revegetation success in the context of provenance and climate change can provide meaningful insights for land managers navigating restoration needs in rapidly changing climate regimes.

Predictive provenancing is a relatively new approach to the issue of revegetation and restoration in a changing climate [39]. The approach matches provenance study results with species distribution models to identify seed sources best-suited for future conditions. While provenance data are available for only a handful of Hawaiian plant species, predictive species distribution models are available for most plants [19]. Comprehensive provenance trials are important for guiding restoration practice, but are not practical investments for the needs of most land managers. General guides outlined by Breed et al. [39] and others (see [11]) coupled with restoration projects that include experimental designs that provide for analysis, even at small scales such as this study, can provide insights when other data are lacking [40].

While overall high seedling survival rates during this study suggest that seedlings can survive in highly exposed conditions despite provenance, invasive species removal efforts were required to reduce competition. A suite of aggressive invasive plants such as matt-forming grasses and bull thistle (*Cirsium vulgare*), lack of native tree canopy, and herbivory by non-native birds presented formidable obstacles to conducting our experiment at KBC. Introduced pasture grasses present perhaps the most pervasive obstacle to restoration of many native ecosystems in Hawai‘i as in other locations [41, 42], reinforcing a trajectory of conversion away from forested environments [43, 44]. Indeed, site protection was one of the only predictors of restoration success in a review by Godefroid et al. [37]. Recent work by Pouteau et al. [45] suggests that while climatic conditions of tropical montane forests will shift upward in elevation, the upslope movement is much less dramatic than previously reported; continued invasion and land use activities may remain the most significant obstacles to restoration success. Quantifying invasive plant regrowth for inclusion as a model predictor may provide understanding of competitive effects on survival and growth in similar future efforts.

Ultimately, the case for understanding future restoration strategies for highly degraded ecosystems is more complicated than predicting native species responses to forecasted climate scenarios [19], because invasive species interactions may present more immediate obstacles [46]. Further experimentation on larger spatial and temporal scales is necessary to determine the empirical responses of species and communities to changing climate in the full context of these biological complexities.

## Supporting information

S1 Table Number of each species that survived by origin and plot at Kanakaleonui Bird Corridor, Hawai‘i Island. Proportion survived is indicated in parentheses.
(PDF)

S2 Table All logistic regression models tested using reverse stepwise variable selection to determine factors influencing survival of *Chenopodium oahuense* seedlings. Model terms included initial seedling height (Initial height), origin of seed (Origin), elevation at which seedling was planted (Elev), temperature range (Temp), soil moisture (Water), and the interaction of Origin with each climate variable: Elev, Temp and Water.
*K* indicates Degrees of freedom. ^*a*^ Indicates full model.
(PDF)

S3 Table All logistic regression models tested using reverse stepwise variable selection to determine factors influencing survival of *Dodonaea viscosa* seedlings. Model terms included initial seedling height (Initial height), origin of seed (Origin), elevation at which seedling was planted (Elev), temperature range (Temp), soil moisture (Water), and the interaction of Origin with each climate variable: Elev, Temp and Water.
*K* indicates Degrees of freedom. ^*a*^ Indicates full model.
(PDF)

S4 Table All logistic regression models tested using reverse stepwise variable selection to determine factors influencing survival of *Sophora chrysophylla* seedlings. Model terms included initial seedling height (Initial height), origin of seed (Origin), elevation at which seedling was planted (Elev), temperature range (Temp), soil moisture (Water), and the interaction of Origin with each climate variable: Elev, Temp and Water.
*K* indicates Degrees of freedom. ^*a*^ Indicates full model.
(PDF)

S5 Table All linear mixed models tested using reverse stepwise variable selection to determine predictors of *Chenopodium oahuense* seedling growth. Model terms included initial seedling height (Initial height), origin of seed (Origin), elevation at which seedling was planted (Elev), temperature range (Temp), soil moisture (Water), and the interaction of Origin with each climate variable: Elev, Temp and Water.
*K* indicates Degrees of freedom. ^*a*^ Indicates full model.
(PDF)

S6 Table All linear mixed models tested using reverse stepwise variable selection to determine predictors of *Dodonaea viscosa* seedling growth. Model terms included initial seedling height (Initial height), origin of seed (Origin), elevation at which seedling was planted (Elev), temperature range (Temp), soil moisture (Water), and the interaction of Origin with each climate variable: Elev, Temp and Water.
*K* indicates Degrees of freedom. ^*a*^ Indicates full model.
(PDF)

S7 Table All linear mixed models tested using reverse stepwise variable selection to determine predictors of *Sophora chrysopholla* seedling growth. Model terms included initial seedling height (Initial height), origin of seed (Origin), elevation at which seedling was planted (Elev), temperature range (Temp), soil moisture (Water), and the interaction of Origin with each climate variable: Elev, Temp and Water.
*K* indicates Degrees of freedom. ^*a*^ Indicates full model.
(PDF)
