# Supplementary material for: Facilitating adaptation to climate change while restoring a montane plant community
Source: PLoS One. 2019 Jun 20;14(6):e0218516. doi: 10.1371/journal.pone.0218516 (PMC6586318; doi:10.1371/journal.pone.0218516)
Supplement: S1 Table — Proportion survived is indicated in parentheses. (PDF) [file pone.0218516.s001.pdf]

| | Species | | | | | |
|---|---|---|---|---|---|---|
| | *C. oahuense* | | *D. viscosa* | | *S. chrysophylla* | |
| Plot elevation (m) | High | Low | High | Low | High | Low |
| 1,927 | 28/28 (1.00) | 26/30 (0.87) | 23/29 (0.79) | 5/8 (0.63) | 5/7 (0.71) | 7/8 (0.88) |
| 1,973 | 16/27 (0.59) | 15/29 (0.52) | 15/29 (0.52) | 6/12 (0.50) | 7/7 (1.00) | 6/8 (0.75) |
| 2,056 | 25/29 (0.86) | 26/28 (0.93) | 24/29 (0.83) | 9/12 (0.75) | 4/7 (0.57) | 6/8 (0.75) |
| 2,095 | 15/28 (0.54) | 21/29 (0.72) | 26/30 (0.87) | 8/12 (0.67) | 6/6 (1.00) | 7/7 (1.00) |
| 2,170 | 27/28 (0.96) | 29/29 (1.00) | 22/29 (0.76) | 9/12 (0.75) | 6/6 (1.00) | 7/8 (0.88) |
| 2,226 | 25/28 (0.89) | 30/30 (1.00) | 27/29 (0.93) | 9/12 (0.75) | 5/7 (0.71) | 5/7 (0.71) |
| 2,276 | 27/29 (0.93) | 29/29 (1.00) | 25/30 (0.83) | 8/12 (0.67) | 5/6 (0.83) | 6/7 (0.86) |
| 2,352 | 15/27 (0.56) | 10/29 (0.34) | 14/29 (0.48) | 0/12 (0.00) | 7/7 (1.00) | 4/7 (0.57) |
| Overall | 178/224 (0.80) | 186/233 (0.80) | 176/234 (0.75) | 54/92 (0.59) | 51/53 (0.96) | 60/67 (0.90) |
