# Supplementary material for: Facilitating adaptation to climate change while restoring a montane plant community
Source: PLoS One. 2019 Jun 20;14(6):e0218516. doi: 10.1371/journal.pone.0218516 (PMC6586318; doi:10.1371/journal.pone.0218516)
Supplement: S2 Table — Model terms included initial seedling height (Initial height), origin of seed (Origin), elevation at which seedling was planted (Elev), temperature range (Temp), soil moisture (Water), and the interaction of Origin with each climate variable: Elev, Temp and Water. K indicates Degrees of freedom. a Indicates full model. (PDF) [file pone.0218516.s002.pdf]

Models	K	AIC_c	Δ AIC_c	w_i
Origin + Elev + Temp + Water + Temp*Origin + (1|Plot)	7	268	0.00	0.31
Origin + Elev + Temp + Water + Elev*Origin + Temp*Origin + Water*Origin + (1|Plot)	9	268	0.36	0.26
Origin + Elev + Temp + Water + Temp*Origin + Water*Origin + (1|Plot)	8	269	0.96	0.19
[a] Initial height + Origin + Elev + Temp + Water + Elev*Origin + Temp*Origin + Water*Origin + (1|Plot)	10	269	1.54	0.14
Origin + Elev + Temp + Water + (1|Plot)	6	271	3.18	0.06
(1|Plot)	2	272	4.28	0.04
